# WS635 Attenuates the Anesthesia/Surgery-Induced Cognitive Impairment in Mice

**DOI:** 10.3389/fnagi.2021.688587

**Published:** 2021-07-21

**Authors:** Jiefu Lin, Fuyi Shen, Jing Lu, Feng Liang, Yiying Zhang, Zhongcong Xie, Yuanlin Dong

**Affiliations:** ^1^Department of Anesthesiology, The First Affiliated Hospital of Jinan University, Guangzhou, China; ^2^Geriatric Anesthesia Research Unit, Department of Anesthesia, Critical Care and Pain Medicine, Massachusetts General Hospital and Harvard Medical School, Charlestown, MA, United States; ^3^Department of Anesthesiology, Shantou Central Hospital, Affiliated Shantou Hospital of Sun Yat-sen University, Shantou, China; ^4^Department of Anesthesiology, Shanghai First Maternity and Infant Hospital, Tongji University School of Medicine, Shanghai, China; ^5^Department of Anesthesiology, Sichuan Provincial People’s Hospital, University of Electronic Science and Technology of China, Chengdu, China

**Keywords:** cognitive impairment, WS635, synapse, ATP, anesthesia/surgery

## Abstract

Anesthesia/surgery has been reported to be associated with perioperative neurocognitive disorder (PND) in patients and induces cognitive impairment in mice. Previous studies demonstrate cyclosporine A (CsA) attenuates the anesthesia/surgery-induced cognitive impairment in mice. However, CsA has immunosuppressive effects and may not be routinely used to prevent or treat PND in patients. WS635 is a nonimmunosuppressive CsA analog. We, therefore, set out to determine whether WS635 could mitigate the anesthesia/surgery-induced cognitive impairment in mice. We performed abdominal surgery under 1.4% isoflurane anesthesia (anesthesia/surgery) for 2 h in 9 month-old wild-type (WT) mice. We treated the mice with CsA (10 mg/kg) or different doses (13.2 mg/kg, 26.4 mg/kg and 52.8 mg/kg) of WS635 before and after the anesthesia/surgery. Barnes maze and fear conditioning system (FCS) were employed to evaluate the cognitive function in mice. We measured the amounts of postsynaptic density (PSD)-95, synaptophysin, and ATP in the hippocampus and cortex of the mice using western blot and ATP Colorimetric/Fluorometric Assay, respectively. We found that the treatment with 52.8 mg/kg, but not 13.2 mg/kg or 26.4 mg/kg, of WS635 attenuated the anesthesia/surgery-induced cognitive impairment in mice and the reductions in the amounts of PSD-95, synaptophysin, and ATP in the mice brain tissues. These results have established a system to study WS635 further and suggest that we need to perform more experiments to determine whether WS635 can ultimately be used as one of the interventions for PND in patients.

## Introduction

Every year, over 312 million patients worldwide have surgery under anesthesia (Weiser et al., 2015). Perioperative neurocognitive disorder (PND; Evered et al., 2018) is one of the most common postoperative complications in these older adults (Inouye et al., 2016; Daiello et al., 2019). It has been reported that PND is associated with Alzheimer’s disease (AD; Fong et al., 2009; Schenning et al., 2016), daily functioning impairments (Phillips-Bute et al., 2006), government assistance dependency (Steinmetz et al., 2009), and increased morbidity and mortality (Monk et al., 2008; Deiner and Silverstein, 2009; reviewed in Vutskits and Xie, 2016). However, there have been no targeted PND interventions, mainly due to the undetermined mechanism of PND.

Our previous studies have shown that abdominal surgery under isoflurane anesthesia can cause cognitive impairment (Yang et al., 2017; Liufu et al., 2020) and delirium-like behavior (Peng et al., 2016; Liufu et al., 2020) in mice. We, therefore, use this established model to study further PND, especially the targeted interventions and underlying mechanism.

Cyclosporine A (CsA) is a blocker of mitochondrial permeability pore (mPTP) opening (Marchetti et al., 1996; Nicolli et al., 1996). Our previous studies found that CsA attenuated the isoflurane-induced mPTP opening, caspase-3 activation, and learning and memory impairment in mice (Zhang et al., 2012). CsA also mitigated the anesthesia/surgery-induced delirium-like behavior in mice (Peng et al., 2016). However, CsA is an immunosuppressive drug in clinics to treat organ rejections and has potential side effects (Penninga et al., 2013; Liddicoat and Lavelle, 2019), limiting its use as an intervention of PND.

WS635, a new compound developed by Waterstone Pharmaceuticals (Wuhan, China), is analogous to CsA without its immunosuppressive effects (Hopkins et al., 2010; Bobardt et al., 2014). WS635 was shown to be orally or intravenous bioavailable in multiple animal species (rat and monkey) and produced blood and liver concentrations of parent drug that exceeded the 50% effective dose determined in the bicistronic con1b-derived replicon assay (Hopkins et al., 2010). However, whether WS635 can be used to prevent or treat PND has not been determined. Therefore, we established a system to further study WS635 by assessing whether WS635 could mitigate the anesthesia/surgery-induced cognitive impairment in mice, using our existing animal model in mice.

Postsynaptic density-95 (PSD-95) is an excitatory postsynaptic marker (Chen et al., 2015; Coley and Gao, 2018), and synaptophysin is a synaptic plasticity-related protein (Janz et al., 1999; Hao et al., 2017; Xiang et al., 2018). Reductions of these synaptic markers suggest synaptic loss, potentially leading to cognitive impairment (Clare et al., 2010; Kaufman et al., 2015; Murmu et al., 2015; Hong et al., 2016). Our lab’s previous study showed that the same anesthesia/surgery induced-cognitive impairment detected in the Barnes maze test and reduced hippocampus levels of PSD-95, synaptophysin, and ATP in brain tissues of mice (Miao et al., 2018). Finally, CsA mitigated the anesthesia/surgery-induced reduction of ATP amounts in mice’s brain tissues (Peng et al., 2016). Therefore, the present study hypothesized that WS635 attenuated the anesthesia/surgery-induced cognitive impairment and reduction in amounts of PSD-95, synaptophysin, and ATP in brain tissues of mice.

PND is a general name for the cognitive impairment identified pre- and postoperative period (Evered et al., 2018). Delayed neurocognitive disorder (dNCR) is one type of PND and could occur about 1–4 weeks after anesthesia/surgery (Evered et al., 2018). In the present study, we determined dNCR in animal studies by assessing the effects of anesthesia/surgery on cognitive function at 2–11 days postoperatively.

## Materials and Methods

### Mice Anesthesia and Surgery Treatment

The Standing Committee on Animals at Massachusetts General Hospital, Boston, MA (protocol 2006N000219) approved the animal protocol. We performed all experiments following the National Institute of Health guidelines and regulations. Efforts were made to minimize the number of animals used. We wrote the manuscript according to ARRIVE guidelines.

Nine-month-old female wild-type (WT) mice (C57BL/J6, Strain#: 000664, Jackson laboratory, Bar Harbor, ME) were randomly assigned to the anesthesia/surgery group or control group by weight. Only female mice were used in the current studies because our previous studies showed that female, but not male, Alzheimer’s disease transgenic mice developed cognitive impairment and synaptic loss following the sevoflurane anesthesia plus surgery (Zhang et al., 2017). All mice were placed in the animal facility to adapt (with 12:12 h light: dark cycle with food and water available ad libitum) for 3 days before the study. The anesthesia/surgery group mice had a simple laparotomy under 1.4% isoflurane in 40% to 100% oxygen using the methods described in our previous studies (Peng et al., 2016; Yang et al., 2017; Miao et al., 2018). 15 min after the induction, the mouse was moved out of the chamber and continuously anesthetized with isoflurane *via* a cone device with one 16-gauge needle inserted into the cone near the mouse’s nose to monitor the concentration of isoflurane. A longitudinal midline incision from the xiphoid to the 0.5-centimeter proximal pubic symphysis was made on the skin, abdominal muscles, and peritoneum. We then sutured the incision layer by layer with 5–0 Vicryl thread. We applied EMLA cream (2.5% lidocaine and 2.5% prilocaine) to the incision site at the end of the procedure and three times per day for 3 days to treat the pain associated with the incision. After the surgical procedure was finished, we put the mouse back into the anesthesia chamber for the rest of the anesthesia up to 2 h. The mice’s rectal temperature was monitored and controlled at 37 ± 0.5°C during the anesthesia/surgery by a feedback-based system with a D.C. Temperature Control System (FHC, Bowdoinham, Maine). The mice were put back to their home cage with food and water available ad libitum after recovering from the anesthesia in 100% O2. The control group mice were placed in their home cages with room air for 2 h, which was consistent with the condition of non-surgery patients. Our previous studies showed that this anesthesia/surgery did not significantly change the mice’s blood pressure and blood gas values (Liufu et al., 2020).

### Treatment With CsA and WS635

CsA (Sigma, St. Louis, MO) was dissolved in corn oil with 10% DMSO. Each of the mice in the control plus CsA group and anesthesia/surgery plus CsA group was injected with CsA solution at the dose of 10 mg/kg in the volume of 0.2 ml through intraperitoneal (IP) at 30 min before, 24 h, and 48 h after the anesthesia/surgery. The mice in the control plus vehicle group and anesthesia/surgery plus vehicle group received 0.2 ml of corn oil with 10% DMSO at the same time points as CsA treatment. WS635 was dissolved in corn oil with 10% DMSO. Each of the mice in control plus WS635 group and anesthesia/surgery plus WS635 group was injected with WS635 solution with dose of 13.2 mg/kg, 26.4 mg/kg, or 52.8 mg/kg in the volume of 0.2 ml through IP at 30 min before, 24 h and 48 h after the anesthesia/surgery. The mice in the vehicle group received 0.2 ml of corn oil with 10% DMSO.

### Measurement of Loss of Right Reflecting (LORR)

The mice received different concentrations of isoflurane, sevoflurane, or desflurane with 52.8 mg/kg WS635 or control condition. Then, the mice were put into the empty home cage, and the cage was rotated 180 degrees to place the mouse on its back. LORR was scored positive if the mouse remained on its back with at least three paws in the air for 60 s.

### Nociception Threshold Determination

We compared the nociception threshold in the mice between the groups of the anesthesia/surgery plus corn oil and the anesthesia/surgery plus WS635 (52.8 mg/kg) at 3 h after the anesthesia/surgery. Nociception threshold was determined by using nylon von Frey filaments as described in a previous study (Chaplan et al., 1994). Precisely, each mouse was placed on a wire mesh platform in clear cylindrical plastic enclosures (8 cm diameter and 10 cm in height). After 20 min of stay on the platform, the filaments were applied (bending force range from 0.008–26 g; North Coast Medical, Morgan Hill, CA) on the incised wound (after the treatment with EMLA) edge and hind paw for 5 s with 10 s between each stimulation. Back arching or withdrawal of the paw from the floor was scored as a positive response, representing the nociception threshold. When no response was obtained, the next stiffer filament in the series was applied to the same area. Each monofilament was used in the test area five times. The nociception threshold was obtained as the force (in grams) at which back arching or paw withdrawal occurred at least three of the five stimulations.

### Barnes Maze

The mice were tested in the Barnes maze as described in a previous study (Liufu et al., 2020). The Barnes maze training was performed from day 7–10 after the anesthesia/surgery. The Barnes maze testing day was day 11 after the anesthesia/surgery. The latency, speed, and distance of the mouse to identify and enter the escape box on training and testing days were measured and recorded with the ANY-Maze video tracking system (Stoelting Co. Wood Dale, IL).

### Fear Conditioning System

The fear conditioning system (FCS) was performed as described by our previous study (Zhang et al., 2012). FCS’s context and tone tests were performed on days 8 and 13 after the anesthesia/surgery. Learning and memory function in both the context and tone tests were assessed by measuring the time the mouse demonstrates “freezing behavior” defined as a completely immobile posture except for respiratory efforts. Any-Maze analyzed the time of such “freezing behavior” (freezing time) (freezing on the threshold: 10; freezing off threshold: 20; minimum freezing duration: 1 s; Stoelting).

### Brain Tissue Harvest, Lysis, and Protein Quantification

The mice’s brain tissues were harvested by decapitation immediately after the anesthesia/surgery. At the end of the FCS, the mice’s brain tissues (cortex and hippocampus) were harvested and stored at −80°C for the western blot analysis. The harvested brain tissues were homogenized on ice using immunoprecipitation buffer (10 mM Tris-HCl, pH 7.4, 150 mM NaCl, 2 mM EDTA, 0.5% Nonidet P-40, Mammalian Protein Extraction Reagent, Thermo Scientific, Waltham, MA) plus protease inhibitors (1 mg/ml aprotinin, 1 mg/ml leupeptin, and 1 mg/ml pepstatin A, Sigma). The lysates were collected, centrifuged at 12,000 rpm for 15 min at 4°C, and quantified for total proteins by bicinchoninic acid (BCA) protein assay kit (Thermo Scientific). The brain tissues were then subjected to western blot analysis.

### Western Blot Analysis

PSD-95 antibody (1:1,000, Cell Signaling, Danvers, MA) was used to detect PSD-95 (95 kDa). Synaptophysin antibody (1:1,000, Abcam, Cambridge, MA) was used to detect synaptophysin (38 kDa). Antibody anti-β-Actin (1:10,000, Sigma) was used to detect β-Actin (42 kDa). Western blot quantification was performed as described by Xie et al. (2008). Briefly, signal intensity was analyzed using ChemiDoc XRS+ with Image Lab 5.0 software (Bio-Rad, Hercules, CA). We quantified Western blots in two steps. First, we used β-Actin levels to normalize (e.g., determine the ratio of PSD-95 to β-Actin amount) the protein amount and to limit the disparities in the protein quantity loaded. Second, we presented the protein amount in the anesthesia/surgery treatment group as a percentage of those in the control group for comparison.

### ATP Measurement

The levels of ATP in the cortex and hippocampus of mice (*N* = 6 in each group) were determined by the ATP Colorimetric/Fluorometric Assay Kit following the protocol provided by the manufacturer (Abcam) as described in the previous studies (Miao et al., 2018).

### Statistical Analysis

Based on our previous studies’ outcomes, we used 5–6 mice per group for the biochemistry studies and 14–18 mice per group for the behavioral studies. We present data from biochemistry studies and FCS freezing time as mean ± standard deviation (SD). Interaction between time and group factors was determined using two-way ANOVA with repeated measurement to analyze the difference in learning curves (based on escape latency) between mice in the control group and the anesthesia/surgery group in the Barnes maze test. Two-way ANOVA and *post hoc* test (Bonferroni correction) were used to analyze the interaction between group (control condition vs. anesthesia/surgery) and treatment (vehicle vs. WS635) on the amounts of PSD-95, synaptophysin, and ATP and freezing time of FCS. The data of nociception threshold were presented as median and interquartile range; and was analyzed by using the Mann–Whitney *U* test. ATP levels were presented as a percentage of those of the control group. *P* values <0.05 were considered statistically significant, and significance testing was two-tailed. Statistical analysis was conducted using GraphPad Prism software (San Diego, CA; version 8.0).

## Results

### Effects of WS635 on the Anesthesia Depth in Mice

**Figure 1:**
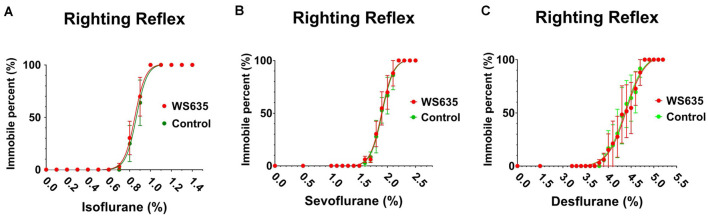
Effects of WS635 on the anesthetic-induced loss of righting reflex in mice. Effects of WS635 and control condition on the presence of loss of righting reflex of mice following different concentrations of isoflurane **(A)**, sevoflurane **(B)**, and desflurane **(C)**. *N* = 6 mice in each group.

First, we asked whether WS635 could alter the anesthesia depth in the mice. The data from the LORR studies demonstrated that WS635 (52.8 mg/kg) did not change the LORR curve of isoflurane (Figure 1A), sevoflurane (Figure 1B), and desflurane (Figure 1C) as compared to the vehicle (the control condition) in the mice. These data suggest that WS635 did not significantly alter the anesthesia depth.

### Effects of WS635 on the Anesthesia/Surgery-Induced Pain

**Figure 2:**
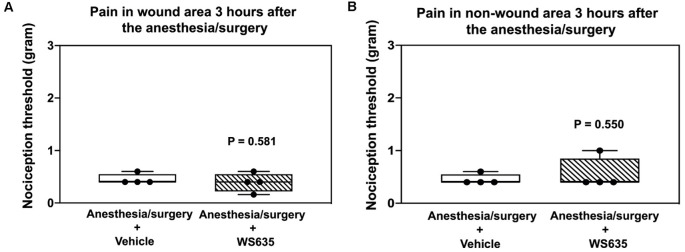
Effects of WS635 on nociception threshold in mice after the anesthesia/surgery. Effects of WS635 and vehicle (corn oil) on the nociception threshold in the wound area **(A)** and non-wound area **(B)** in the mice following the anesthesia/surgery. WS635 did not significantly alter the nociception threshold compared to the vehicle in the mice following the anesthesia/surgery. *N* = 4 mice in each group.

We asked whether WS635 could alter the nociception threshold in the mice after the anesthesia/surgery. The data from the nociception threshold studies demonstrated that WS635 (52.8 mg/kg) did not significantly change the nociception threshold in the wound area (Figure 2A) and non-wound area (Figure 2B) as compared to the vehicle (corn oil) in the mice. Notably, we put EMLA in the wound area before the measurement of the nociception threshold. These data suggest that WS635 did not significantly alter the nociception threshold in both wound and non-wound areas of the mice following the anesthesia/surgery.

### Effects of WS635 on the Locomotor Activity of the Mice

**Figure 3:**
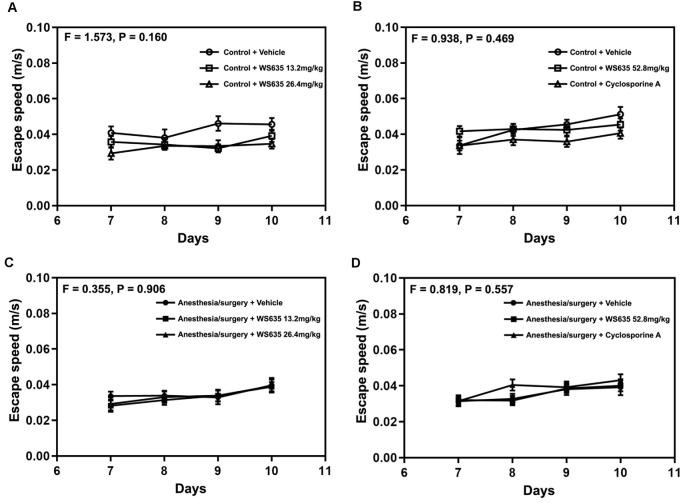
Effects of WS635, CsA, and the anesthesia/surgery on locomotor activity in mice. Effects of WS365 with 13.2 mg/kg and 26.4 mg/kg **(A–C)** and effects of WS365 with 52.8 mg/kg and CsA with 10 mg/kg **(B–D)** on escape speed of Barnes maze on training days (7–10 days after the anesthesia/surgery) in mice following control condition or anesthesia/surgery. *N* = 14–18 mice in each group.

Next, we found that the treatment with 13.2 mg/kg or 26.4 mg/kg of WS635 did not significantly change the escape speed as compared to the vehicle in the control condition (Figure 3A) or the anesthesia/surgery condition (Figure 3C) in the mice tested in Barnes maze. Moreover, the treatment with 52.8 mg/kg of WS635 or 10 mg/kg of CsA did not significantly change the escape speed compared to the vehicle treatment in either control condition (Figure 3B) or anesthesia/surgery condition (Figure 3D) in the mice. These data suggest that WS635 did not significantly alter the locomotor activity of the mice.

### WS635 Attenuated the Anesthesia/Surgery-Induced Cognitive Impairment in Mice

**Figure 4:**
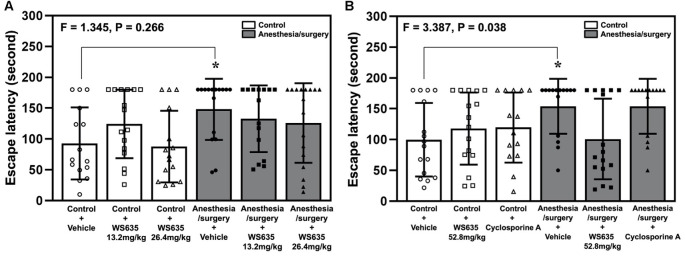
Effects of WS635, CsA, and the anesthesia/surgery on cognitive function in mice detected in Barnes maze. **(A)** The effects of WS635 with 13.2 mg/kg and 26.4 mg/kg on the anesthesia/surgery-induced changes in Barnes maze’s escape latency at testing day (11 days after the anesthesia/surgery) in mice. **(B)** The effects of WS635 with 52.8 mg/kg and CsA on the anesthesia/surgery-induced changes in Barnes maze’s escape latency at testing day (11 days after the anesthesia/surgery) in mice. *N* = 14–17 mice in each group. Two-way ANOVA with the *post hoc* Bonferroni correction was used. *P < 0.05. *P* values refer to the difference among different conditions on the escape latency. CsA, cyclosporine A.

The anesthesia/surgery increased the Barnes maze’s escape latency on the testing day compared to the control condition (Figure 4A). The treatment with 13.2 mg/kg or 26.4 mg/kg of WS635 did not significantly attenuate the anesthesia/surgery-induced increases in the escape latency (Figure 4A). However, two-way ANOVA showed a significant interaction of treatment (vehicle, 52.8mg/kg WS635 and CsA) and group (control condition vs. anesthesia/surgery) on the escape latency of Barnes maze on the testing day (*F* = 3.387, *P* = 0.038, Figure 4B). Specifically, the treatment with 52.8 mg/kg of WS635 significantly attenuated the anesthesia/surgery-induced increases in the Barnes maze’s escape latency on the testing day (11 days after the anesthesia/surgery) compared to the vehicle (*P* = 0.037, Figure 4B).

**Figure 5:**
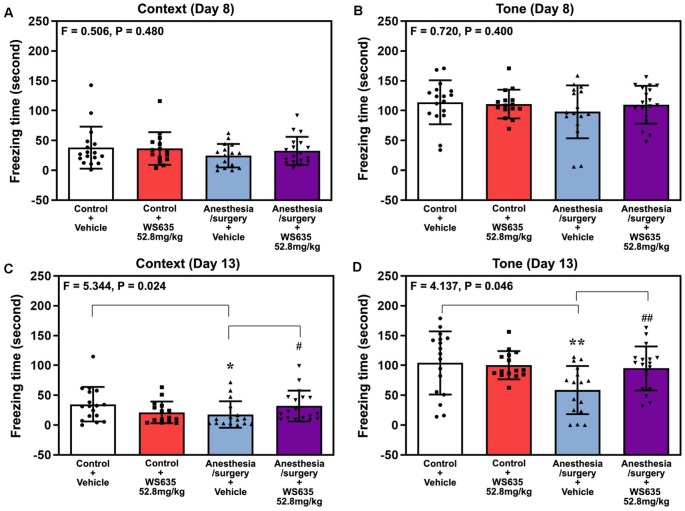
Effects of WS635 on the anesthesia/surgery-induced changes in the cognitive function in mice detected in fear conditioning system (FCS). Effects of WS365 (52.8 mg/kg) and vehicle (DMSO) on the anesthesia/surgery-induced changes in the freezing time on the context **(A)** and tone **(B)** test of FCS on 8 days after the anesthesia/surgery; and on the context **(C)** and tone **(D)** test of FCS on 13 days after the anesthesia/surgery. *N* = 14–18 mice in each group. Two-way ANOVA with the *post hoc* Bonferroni correction was used. * or ^#^*P* < 0.05; ** or ^##^*P* < 0.01. *P* values refer to the difference among different conditions on the freezing time of the FCS. DMSO, dimethyl sulfoxide; FCS, fear conditioning system.

Next, we repeated the study in the FCS. The anesthesia/surgery did not significantly change mice’s freezing time in the context test (Figure 5A) or the tone test (Figure 5B) of the FCS on day 8 after the anesthesia/surgery. However, two-way ANOVA showed significant interaction of treatment (vehicle vs. 52.8 mg/kg of WS635) and group (control condition vs. anesthesia/surgery) in the context test (*F* = 5.344, *P* = 0.024, Figure 5C) and tone test (*F* = 4.137, *P* = 0.046, Figure 5D) of the FCS on 13 days after the anesthesia/surgery. Specifically, the anesthesia/surgery significantly reduced mice’s freezing time in the context (*P* = 0.038, Figure 5C) and tone tests (*P* = 0.009, Figure 5D) of the FCS as compared to control condition in the mice on day 13 after the anesthesia/surgery. The treatment with 52.8 mg/kg of WS635 attenuated the anesthesia/surgery-induced decreases in the freezing time in the context (*P* = 0.024, Figure 5C) and tone (*P* = 0.046, Figure 5D) tests of the FCS on day 13 after the anesthesia/surgery as compared to vehicle in the mice. Taken together, the data suggest that WS635 attenuates the anesthesia/surgery-induced cognitive impairment in the mice in a dose-dependent manner.

### WS635 Attenuated the Anesthesia/Surgery-Induced Reductions in Synaptic Markers in Brain Tissues of Mice

**Figure 6:**
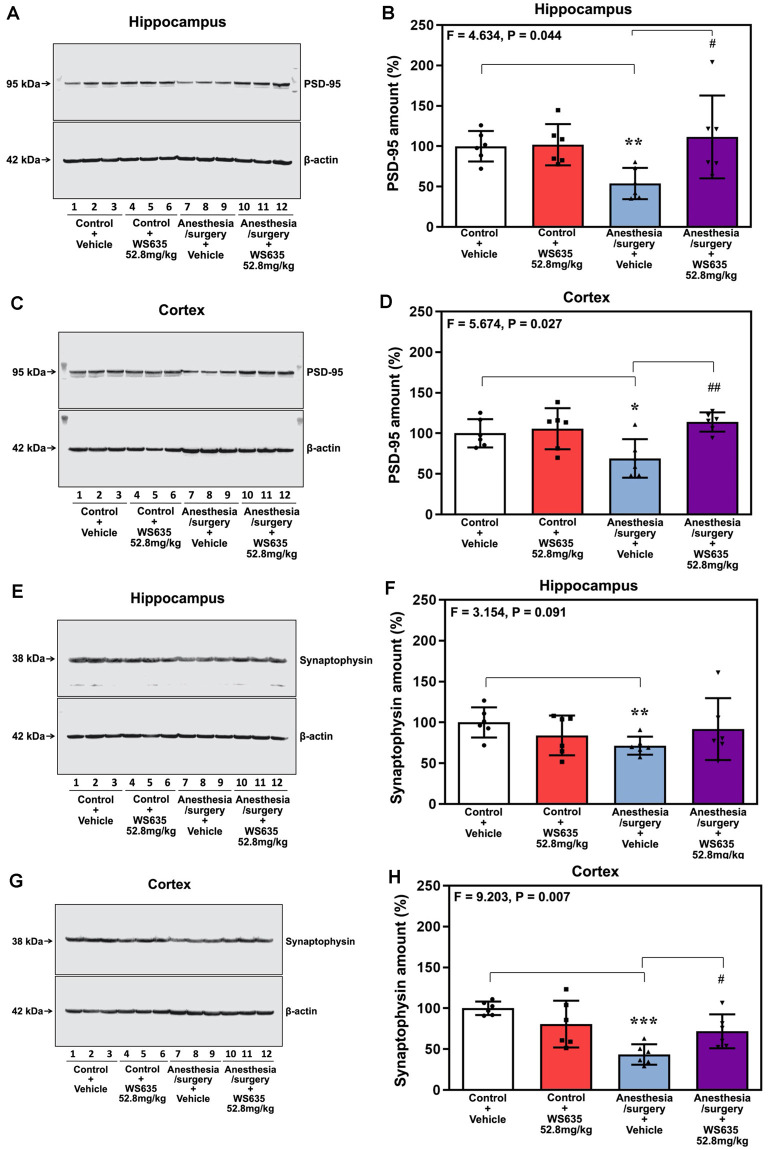
Effects of WS635 on the anesthesia/surgery-induced changes in the amounts of postsynaptic density (PSD)-95 and synaptophysin in the hippocampus and cortex of mice. Effects of WS365 (52.8 mg/kg) and vehicle (DMSO) on the anesthesia/surgery-induced changes in the amounts of PSD-95 in the hippocampus **(A,B)** and cortex **(C,D)** of the mice on day 13 after the anesthesia/surgery. Effects of WS365 (52.8 mg/kg) and vehicle (DMSO) on the anesthesia/surgery-induced changes in the amounts of synaptophysin in the hippocampus **(E,F)** and cortex **(G,H)** of the mice on day 13 after the anesthesia/surgery. *N* = 6 mice in each group, * or ^#^*P* < 0.05; ** or ^##^*P* < 0.01; *** *P* < 0.001. Two-way ANOVA with the *post hoc* test (Bonferroni correction) was used. *P* values refer to the difference among different conditions on the amounts of the PSD-95 and synaptophysin. DMSO, dimethyl sulfoxide.

Given the findings that WS635 attenuated the anesthesia/surgery-induced cognitive impairment in the mice, we asked whether WS635 could mitigate the anesthesia/surgery-induced reductions in the synaptic markers in mice brain tissues. Quantitative western blot analysis showed that the anesthesia/surgery induced a visible decrease of the amounts of PSD-95 in both hippocampi (Figure 6A) and cortex (Figure 6C). Two-way ANOVA showed significant interaction of treatment (vehicle vs. WS635) and group (control condition vs. anesthesia/surgery) on the amounts of PSD-95 in the hippocampus (*F* = 4.634, *P* = 0.044, Figure 6B) and cortex (*F* = 5.674, *P* = 0.027, Figure 6D). Specifically, WS635 mitigated the anesthesia/surgery-induced reduction in PSD-95 amounts in the hippocampus (Figure 6B) and cortex (Figure 6D). Quantitative western blot also showed that WS635 mitigated the anesthesia/surgery-induced reductions in synaptophysin amounts in the hippocampus (*P* = 0.091, borderline significance, Figures 6E,F) and cortex (*P* = 0.007, Figures 6G,H). Given that the molecular weights of synaptophysin (38 kDa) and β-actin (42 kDa) are similar, we used two, instead of one, gels from the same experimental samples with the same loading amounts performed at the same time to present the bands of synaptophysin and β-actin, respectively, in Figures 6E–G. These data suggest that WS635 can attenuate the anesthesia/surgery-induced cognitive impairment *via* mitigating the anesthesia/surgery-induced reduction in the synapse.

### Effects of WS635 on Mouse Brain ATP Amounts After Anesthesia/Surgery

**Figure 7:**
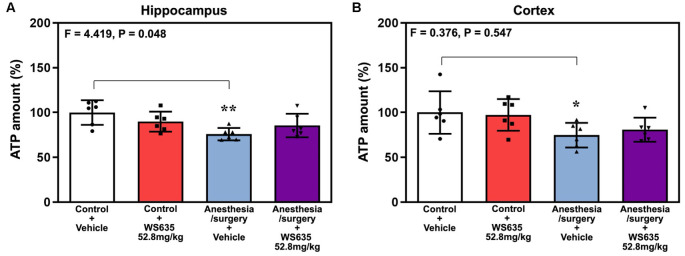
Effects of WS635 on the anesthesia/surgery-induced changes in ATP amounts in the hippocampus and cortex of mice. Effects of WS365 (52.8 mg/kg) and vehicle (DMSO) on the anesthesia/surgery-induced changes in the amounts of ATP in the hippocampus **(A)** and cortex **(B)** of the mice immediately after the anesthesia/surgery. *N* = 6 mice in each group, * *P* < 0.05; ** *P* < 0.01. Two-way ANOVA with the *post hoc* test (Bonferroni correction) was used. *P* values refer to the difference among different conditions on the amounts of ATP. DMSO, dimethyl sulfoxide.

Anesthesia/surgery decreased ATP amounts in the hippocampus (Figure 7A) and cortex (Figure 7B) of the mice. Two-way ANOVA showed a significant interaction of treatment (vehicle vs. WS635) and group (control condition vs. anesthesia/surgery) on the ATP amounts in the hippocampus (Figure 7A) but not in the cortex (Figure 7B) of mice. These data suggest that WS635 mitigates the anesthesia/surgery-induced reduction in ATP amounts in mice hippocampus.

## Discussion

This pilot preclinical study established a system and explored whether WS635, a novel compound and an analog of CsA, could be an intervention of postoperative cognitive impairment in mice. The findings that WS635 mitigated the anesthesia/surgery-induced cognitive impairment in mice suggest that we should perform more studies to determine whether WS635 is an effective intervention of cognitive impairment in rodents and PND in patients.

We first demonstrated that WS635 did not alter the LORR of mice following different concentrations of isoflurane, sevoflurane, and desflurane (Figure 1). WS635 did not significantly change the nociception threshold (Figure 2) nor the escape speed of the mice (Figure 3). These data suggest that WS635 may not significantly affect the depth of anesthesia and may not change the locomotor activity nor nociception threshold in mice. Then, we found that the treatments with 13.2 mg/kg or 26.4 mg/kg of WS635 did not mitigate the anesthesia/surgery-induced increases in escape latency in the mice tested in the Barnes maze. However, the treatment with 52.8 mg/kg of WS635 attenuated the anesthesia/surgery-induced increases in escape latency in the mice tested in the Barnes maze (Figure 4). These data suggest that WS635 can mitigate the anesthesia/surgery-induced cognitive impairment in mice dose-dependently. Moreover, the treatment with 52.8 mg/kg WS635 mitigated the anesthesia/surgery-induced decreases in freezing time of mice tested in FCS (Figure 5), further suggesting that WS635 can treat the anesthesia/surgery-induced cognitive impairment in the mice. Consistently, the treatment with 52.8 mg/kg WS635 mitigated the anesthesia/surgery-induced reduction in the amounts of PSD-95 and synaptophysin in the hippocampus and cortex of mice (Figure 6). These data suggest that WS635 may mitigate the anesthesia/surgery-induced cognitive impairment by attenuating the anesthesia/surgery-induced synaptic loss. Future studies to test this hypothesis are warranted.

There are few studies to investigate WS635 so far. The only pharmacokinetic study of WS635 was performed in rats and monkeys to measure the amounts of WS635 in blood and liver following oral or intravenous administration of WS635 (Hopkins et al., 2010). In the present system establishment study in mice, we used intraperitoneal (IP) administration of WS635 because it is generally more difficult to perform oral or intravenous administration than IP administration in mice. We did not perform a bioavailability experiment in this proof of concept study. However, the findings that the IP administration of WS635 attenuated the anesthesia/surgery-induced behavioral and biochemistry changes suggest the effectiveness of WS635 following the IP administration. It remains unknown whether WS635 can reach brain tissues after administration. WS635 could attenuate the effects of the anesthesia/surgery on cognitive function and brain amounts of ATP and synaptic markers through the target(s) in blood, peripheral nervous system, or central nervous system. However, the exact target(s) of WS635, e.g., central nervous system vs. peripheral nervous system vs. blood, remains undetermined at present. Nevertheless, we will use the established system to further study WS635, including systematic pharmacokinetic and mechanistic investigations, in the future.

Interestingly, WS635 only mitigated the anesthesia/surgery-induced reduction in ATP amounts in the hippocampus but not the mice’s cortex (Figure 7). These data suggest that WS635 may not fully regulate the mitochondrial function of the mice’s brain tissues. Therefore, we postulate that WS635 can improve the mitochondrial function of both peripheral and brain tissues, leading to the mitigation of the anesthesia/surgery-induced cognitive impairment. A future study to identify the target of WS635 action is warranted.

There have been no studies to determine whether WS635 can regulate cognitive function in rodents. We, therefore, cannot compare the data obtained from the current study and the data obtained from previous studies. However, previous studies showed that idebenone could mitigate the anesthesia-induced reduction in mouse brain ATP amounts and the anesthesia-induced cognitive impairment in mice (Zhang et al., 2020). Idebenone is a synthetic analog of coenzyme Q (Rauchova et al., 2008). It has been reported that idebenone can protect mitochondrial function (Orsucci et al., 2011; Erb et al., 2012; reviewed in Pfeffer et al., 2013). Idebenone can cross the blood-brain barrier (Torii et al., 1985; Nagai et al., 1989). These findings suggest that it is important to determine whether WS635 can cross the blood-brain barrier and identify the target (peripheral vs. brain tissues) of WS635 action.

One study has shown that short-term administration of CsA can attenuate the APOE4-mediated neurovascular injury, including a breakdown of the blood-brain barrier, neuronal dysfunction, and neurodegenerative changes (Bell et al., 2012). Another study demonstrates that loss of low-density lipoprotein receptor-related protein 1 leads to the breakdown of the blood-brain barrier, neuronal loss, and cognitive impairment *via* cyclophilin A-matrix metalloproteinase-9 pathway in the endothelium (Nikolakopoulo et al., 2021). These data further suggest the role of mitochondrial and CsA in cognitive impairment and neurodegeneration. Our previous studies have also demonstrated that short-term administration of CsA can attenuate the anesthesia-induced mitochondrial dysfunction and cognitive impairment *in vitro* and in mice (Zhang et al., 2012) and the anesthesia/surgery-induced reduction in brain ATP amounts, the increases in latency to eat food in buried food test, and the decreases in entries in the novel arm in mice in *Y* maze test (Peng et al., 2016). These results suggest that short-term administration of CsA could be a promising treatment for postoperative cognitive impairment. However, the present study only focused on establishing a system to investigate WS635. Therefore, we did not systematically study the effects of CsA. We will use the established system to compare the effects of CsA and WS635 in future pre-clinical studies and potential clinical investigations.

There has been no reference regarding the dosage of WS635 we could use to determine the effects of WS635 on cognitive function in mice. WS635 is not commercially available and was exclusively provided by Waterstone Pharmaceuticals *via* Material Transfer Agreement. After consulting with Waterstone Pharmaceuticals, we decided to start our studies using WS635 with the dosage of 13.2, 26.4, and 52.8 mg/kg in mice. The lower dosage (13.2 and 26.4 mg/kg) of WS635 did not attenuate the anesthesia/surgery-induced changes in mice. We, therefore, used 52.8 mg/kg of WS635 in the study, which attenuated the anesthesia/surgery-induced cognitive impairment, ATP reduction, and synaptic loss without mortality or apparent abnormal behaviors observed in the mice.

There were several limitations in this study. First, we do not have methods and technology to determine whether WS635 can cross the blood-brain barrier without anesthesia/surgery. Therefore, we do not know the exact targets and mechanisms by which WS635 mitigates the anesthesia/surgery-induced cognitive impairment in mice and the reductions in synaptic markers and ATP in mice brain tissues. Second, we performed various behavioral tests and biochemistry tests at different times after the anesthesia/surgery. These times were chosen based on the findings from our previous studies (Yang et al., 2017) and the fact we targeted dNCR (occurring between 1–4 weeks after anesthesia/surgery) in the present study, which could lead to missing the effects of anesthesia/surgery on earlier (e.g., 1–3 days) cognitive function. However, the present study’s main objective was to establish a system and prove a concept that WS635 could mitigate anesthesia/surgery-induced cognitive impairment in mice. We will use the established approach to identify the underlying mechanism and time course of WS635 in the future. Finally, we did not measure the effects of WS635 on liver function, leakage of the blood-brain barrier, microglial activation, neuronal or axonal degeneration, among others in the mice. However, performing these experiments would take a long time and significantly delay the publication of the current findings that focused on establishing a system and proof of a concept. Therefore, we will perform these studies in the future.

In conclusion, we have established a system to study WS635 in mice and found that WS635 attenuated the cognitive impairment induced by anesthesia/surgery in a dose-dependent way. The treatment of WS635 attenuated the anesthesia/surgery-induced synaptic loss, and reduction in ATP amounts, in brain tissues of mice. These findings will promote more research of WS635, ultimately leading to identifying the underlying mechanism and revealing targeted interventions of PND in patients.

## Data Availability Statement

The original contributions presented in the study are included in the article, further inquiries can be directed to the corresponding author/s.

## Ethics Statement

The animal study was reviewed and approved by The Standing Committee on Animals at Massachusetts General Hospital, Boston, MA (protocol 2006N000219).

## Author Contributions

Experimental design: YD, FL, YZ, and ZX. Performance of the experiments: JLin, FS, and JLu. Data analysis: JLin, FS, JLu, and YD. Manuscript writing: JLin, YD, and ZX.

## Conflict of Interest

The authors declare that the research was conducted in the absence of any commercial or financial relationships that could be construed as a potential conflict of interest.
